# Adjunctive Use of Platelet‐Rich Plasma With Fractional CO_2_ Laser Versus Standalone Laser Therapy for the Treatment of Atrophic Acne Scarring: A Systematic Review and Meta‐Analysis

**DOI:** 10.1111/jocd.71141

**Published:** 2026-09-10

**Authors:** Qiang Hu, Shuang Sui, Zhenghua Yang, Kaiwen Zhang, Haibo Wang

**Affiliations:** ^1^ Department of Burn and Plastic Surgery The 970th Hospital of the Joint Service Support Force of the Chinese People's Liberation Army Yantai Shandong China; ^2^ Department of Gastrointestinal Surgery The 970th Hospital of the Joint Service Support Force of the Chinese People's Liberation Army Yantai Shandong China

**Keywords:** atrophic acne scars, combination therapy, fractional CO_2_ laser, meta‐analysis, platelet‐rich plasma, randomized controlled trials

## Abstract

**Background:**

Atrophic acne scars are a common and persistent sequela of inflammatory acne. Fractional CO_2_ laser treatment promotes collagen production and scar remodeling but is often associated with adverse effects such as prolonged erythema, edema, and hyperpigmentation. Adjunctive use of platelet‐rich plasma (PRP) has been proposed to enhance efficacy and reduce complications, yet existing evidence remains inconsistent.

**Methods:**

This meta‐analysis of randomized controlled trials (RCTs) compared PRP combined with fractional CO_2_ laser versus laser alone for atrophic acne scars. Following PRISMA methodology, databases were searched up to July 31, 2025. Data were synthesized using RevMan 5.4 and R 4.5.2. Seven RCTs involving 165 patients were included.

**Results:**

Combination therapy significantly outperformed laser monotherapy in excellent improvement rate (> 75%; OR = 3.21, 95% CI: 1.04–9.83, *p* = 0.04) and patient satisfaction (very satisfied; OR = 3.05, 95% CI: 1.27–7.29, *p* = 0.01). With respect to safety outcomes, no statistically significant differences were observed between the two groups in duration of erythema, duration of edema, incidence of hyperpigmentation, or frequency of persistent erythema (all *p* > 0.05), indicating that adding PRP did not increase postoperative adverse events.

**Conclusion:**

Current evidence, though limited by small sample size, short follow‐up (≤ 6 months in all included trials), and a study population restricted predominantly to Middle Eastern and East Asian skin types (Fitzpatrick III–V), suggests that combining PRP with fractional CO_2_ laser may improve short‐term efficacy and patient satisfaction compared with laser alone, while maintaining a comparable safety profile. Larger, well‐designed RCTs with extended follow‐up (≥ 12 months) and broader skin types (I–II and VI) are warranted.

**Trial Registration:**

International Prospective Register of Systematic Reviews (PROSPERO) identification number: CRD420261283574

## 1 Introduction

Among the long‐term consequences of inflammatory acne, atrophic scarring is considered the most frequently encountered and enduring complication. Their pathogenesis stems from the destruction and impaired repair of key dermal matrix components, particularly collagen and elastic fibers, following inflammatory injury, which leads to tissue depression and abnormal texture [1]. Affecting up to 95% of acne patients, such scarring adversely affects mental health and daily functioning. Fractional CO_2_ laser treatment, which promotes collagen production and structural remodeling through focal photothermal effects, has become a mainstay in physical scar revision strategies [2, 3, 4]. Given that this technique relies on inducing controlled dermal injury, it frequently results in undesirable effects such as erythema, edema, crust formation, and post‐inflammatory hyperpigmentation. These adverse events, to some extent, limit its widespread adoption and diminish patient satisfaction with the treatment.

To enhance therapeutic efficacy and minimize adverse effects, platelet‐rich plasma (PRP) has been introduced as a biological adjunct in combination treatment regimens [5]. This autologous preparation serves as a concentrated source of platelet‐derived growth factors, transforming growth factor‐β, and a range of other cytokines. These bioactive molecules help regulate inflammation, promote angiogenesis, stimulate fibroblast activity, and facilitate collagen deposition [6]. The microchannels generated by fractional CO_2_ laser ablation are hypothesized to facilitate enhanced percutaneous penetration of PRP's bioactive constituents, thereby enabling synergistic interaction. Concurrently, PRP may optimize the wound microenvironment following laser therapy, thereby accelerating repair and reducing the risk of complications [7, 8].

Although some evidence supports the benefits of combination therapy for atrophic acne scars [9, 10], existing studies exhibit significant heterogeneity. Variations in PRP preparation and administration methods (injection versus topical application), laser parameters, outcome measures, and follow‐up durations have led to inconsistent conclusions [11, 12]. Furthermore, previous systematic reviews were limited by methodological differences and inclusion of non‐randomized studies, without fully exploring key factors such as PRP delivery methods.

Therefore, this systematic review and meta‐analysis, strictly adhering to PRISMA guidelines, aims to evaluate the efficacy and safety of fractional CO_2_ laser combined with PRP therapy for atrophic acne scars. Unlike previous meta‐analyses, this review includes only randomized controlled trials, applies the RoB 2 tool for risk of bias assessment, and systematically evaluates PRP preparation protocols, thereby providing more reliable evidence for clinical practice.

## 2 Materials and Methods

This systematic review and meta‐analysis was prospectively registered with the International Prospective Register of Systematic Reviews (PROSPERO). The execution and reporting of this review will strictly adhere to the Preferred Reporting Items for Systematic Reviews and Meta‐Analyses (PRISMA) guidelines [13].

### 2.1 Literature Search Strategy

Two information specialists independently performed a systematic search of the literature across four electronic databases, including PubMed, Embase, Web of Science, and the Cochrane Library (with a focus on the Cochrane Central Register of Controlled Trials, CENTRAL). The search covered the period from the inception of each database up to July 31, 2025. Search strategies were formulated by combining controlled vocabulary terms with free‐text keywords and were tailored to the syntax of each database. As an example, the following search strategy was applied in PubMed: (“Platelet‐Rich Plasma” [Mesh] OR “platelet rich plasma” [tiab] OR PRP[tiab]) AND (“Acne Vulgaris” [Mesh] OR acne[tiab]) AND (“Cicatrix” [Mesh] OR scar [tiab] OR atrophic scar [tiab]) AND (“Lasers, Gas” [Mesh] OR “Carbon Dioxide” [Mesh] OR “fractional CO_2_ laser” [tiab] OR “CO_2_ laser” [tiab]) AND (“Randomized Controlled Trial” [pt] OR randomized [tiab] OR randomised [tiab] OR placebo [tiab]). In addition to database searches, we manually examined reference lists of relevant systematic reviews and included studies, as well as pertinent conference proceedings. Where studies met inclusion criteria but reported incomplete data, corresponding authors were contacted by email in an effort to obtain the necessary raw information. Full search strategies for all databases are provided in Supporting Information.

### 2.2 Inclusion and Exclusion Criteria

The eligibility criteria for selecting studies in this review were defined as follows: (1) Population (P): Patients clinically diagnosed with atrophic acne scars; (2) Intervention (I): The use of autologous PRP in conjunction with fractional CO_2_ laser therapy; (3) Comparison (C): Treatment with fractional CO_2_ laser monotherapy; (4) Outcomes (O): Documentation of a minimum of one primary or secondary endpoint related to the investigation's outcomes; and (5) Study design (S): Randomized controlled trials (RCTs) employing either parallel‐group or self‐controlled (e.g., split‐face) designs were eligible. Studies were deemed ineligible and subsequently removed based on the following exclusion criteria: (1) publication in languages other than English; (2) unavailability of full text or absence of critical outcome data; (3) duplicate publications; and (4) non‐randomized controlled trial methodologies.

### 2.3 Literature Screening and Data Extraction

The tasks of screening the literature and extracting data were carried out independently by two reviewers. After uploading the search results into EndNote X9 and removing duplicate entries, each reviewer independently assessed the titles and abstracts of the remaining studies to exclude those clearly irrelevant. For studies deemed potentially relevant, full‐text versions were retrieved and reassessed against the established inclusion and exclusion criteria to confirm their eligibility. Any disagreements arising during the study selection process were addressed either through deliberative consensus between the two reviewers or by involving an additional reviewer as an arbitrator. A predefined digital data extraction sheet was employed to systematically gather information covering: (1) Study characteristics (including authors, publication year, country of origin, study design, sample size, and follow‐up duration); (2) Patient characteristics (comprising mean age, sex distribution, Fitzpatrick skin phototype, and baseline severity of scarring); (3) Intervention details (laser device and parameters, number of treatments, PRP preparation method and application); (4) Outcome measure data, including efficacy outcomes (excellent improvement rate), safety outcomes (erythema duration, edema duration, incidence of hyperpigmentation, incidence of persistent erythema), and patient satisfaction rate. For continuous variables, data on the mean, standard deviation, and sample sizes per group were obtained from the primary studies. For dichotomous variables, the event count and total number of participants were recorded.

Operational definitions of the main outcomes were as follows: excellent improvement rate was defined as > 75% improvement in scar appearance assessed by blinded physicians using a quartile grading scale (poor: < 25%, mild: 25%–49%, moderate: 50%–74%, excellent: > 75%); patient satisfaction rate was defined as the proportion of patients rating themselves as “very satisfied” on a 4‐point scale; persistent erythema was defined as erythema lasting more than 7 days after treatment or as reported by the original investigators; hyperpigmentation was defined as post‐inflammatory hyperpigmentation at the treated sites assessed clinically.

### 2.4 Risk of Bias Assessment

Two reviewers independently assessed the risk of bias of the included RCTs using the Cochrane RoB 2 tool. Five key domains were systematically evaluated: the process of randomization, adherence to the assigned interventions, the completeness of outcome data, the methods used for outcome measurement, and the transparency of outcome reporting. Following this evaluation, each trial was categorized as having an overall judgment of “low risk”, “some concerns”, or “high risk” of bias. In instances where the two reviewers arrived at differing judgments, agreement was achieved through discussion.

### 2.5 Statistical Analysis

All data analyses were carried out using RevMan 5.4 and R 4.5.2. For continuous outcome measures, treatment effects were estimated using mean differences or standardized mean differences, each accompanied by 95% confidence intervals. Dichotomous data were summarized using odds ratios with their corresponding 95% confidence intervals. Inter‐study heterogeneity was assessed by means of Cochran's *Q* test (with statistical significance defined as *α* = 0.10) and the *I* ^2^ statistic. A fixed‐effects model was applied in cases of low heterogeneity (*p* ≥ 0.10 and *I* ^2^ ≤ 50%); conversely, a random‐effects model was employed when substantial heterogeneity was detected (*p* < 0.10 or *I* ^2^ > 50%), followed by subgroup analyses aimed at investigating potential sources of variability. Predefined subgroup analyses were conducted according to PRP delivery method, skin phototype, and treatment frequency, with differences between subgroups formally tested. Sensitivity analyses involved sequentially removing each study to examine the stability of results. The impact of excluding the study that received a “high risk” judgment in any domain of the RoB 2 tool was specifically examined. All included studies were retained for the primary meta‐analysis regardless of their risk of bias ratings. If at least 10 studies contributed to an outcome, funnel plots and Egger's test would be used to assess publication bias. The GRADE approach was applied to rate the certainty of evidence for primary efficacy and safety outcomes. Six of the seven included trials employed a split‐face design, and one trial used a three‐arm parallel design. None of the split‐face trials reported individual patient‐level paired data; therefore, all analyses were performed using aggregate group‐level data with standard meta‐analytic methods, and the model selection criteria described above were applied.

## 3 Results

### 3.1 Characteristics of the Included Studies

**FIGURE 1:**
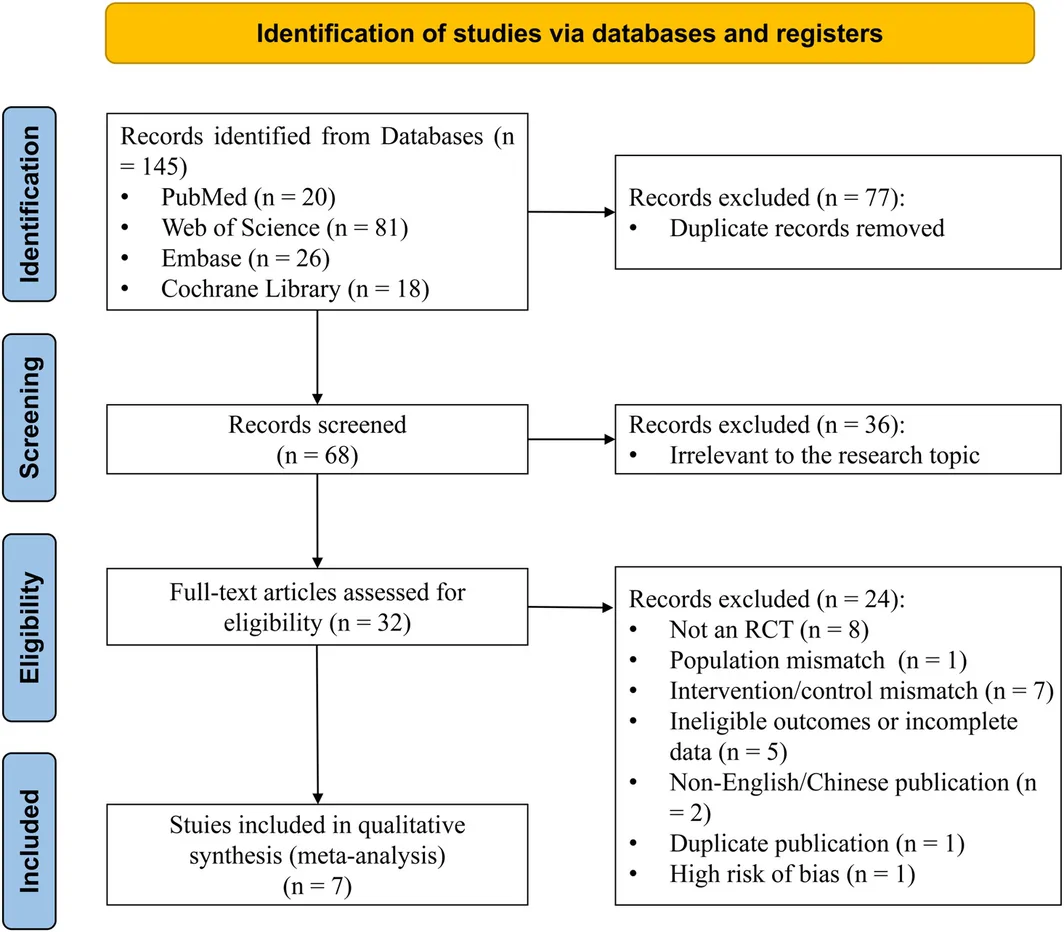
PRISMA flow diagram of study selection for the systematic review and meta‐analysis.

The search identified 145 records. Following the exclusion of 77 duplicate entries and a two‐phase selection process involving screening of titles and abstracts along with full‐text evaluation, conducted in accordance with PRISMA guidelines (Figure 1), a total of seven RCTs were deemed eligible for inclusion in both qualitative synthesis and quantitative meta‐analysis [14, 15, 16, 17, 18, 19, 20]. These 7 studies enrolled a total of 165 patients. In terms of geographic distribution, the included trials originated predominantly from Egypt (three studies) and Korea (two studies), with single studies contributed by Iran and India. Six of the seven included trials employed a split‐face design, while one three‐arm parallel trial (El‐Hawary 2020) was also included. All trials recruited individuals diagnosed with moderate to severe atrophic acne scars, spanning Fitzpatrick skin phototypes II through V. A detailed overview of the core characteristics of these studies, including the specific therapeutic protocols, control conditions, outcome evaluation techniques, and observation periods, is presented in Table 1.

**TABLE 1:** Summary of study characteristics from included randomized controlled trials.

Study	Country	Study design	Sample size (*n*)	Patient characteristics	Laser parameters	PRP preparation & application	Control	Treatment sessions & interval	Outcome measures	Follow‐up	Scar grading system
Lee (2011) [14]	South Korea	Randomized, split‐face	14	Skin Types III–V; moderate to severe atrophic acne scars	Pulse energy: 25 mJ/150 μm MTZ; density: 400 MTZ/cm^2^; forced‐air cooling	Double‐spin (3000 rpm/3 min → 4000 rpm/3 min); CaCl_2_ activation; intradermal injection (0.3 mL/site, 1.5–2 cm apart)	Normal saline intradermal	2 sessions; 4‐week interval	e, f	4 months after last session	Quartile grading scale (0–4)
Faghihi et al., (2016) [15]	Iran	Randomized, split‐face	16	Skin Types II–IV; moderate to severe atrophic acne scars	Power: 25; dot cycle: 3; energy: 30 mJ; pitch: 1; ablation depth: 600 μm	Double‐spin (2000 g/3 min → 5000 g/5 min); CaCl_2_ activation; intradermal injection (0.2 mL/site, 2 cm apart)	Normal saline intradermal	2 sessions; 4‐week interval	d	4 months after last session	Quartile grading scale (0–3) for patient satisfaction
Abdel Aal et al. (2017) [16]	Egypt	Single‐blind, split‐face	30	Skin Types III–V; atrophic acne scars (boxcar, ice‐pick, rolling)	Power: 15 W; dwell time: 600 μs; spacing: 700 μm; stack level: 3	Double‐spin (3000 rpm/7 min → 4000 rpm/5 min); CaCl_2_ activation (1:9); intradermal injection (0.1 mL/site, 1–1.5 cm apart)	No injection (laser alone)	2 sessions; 3–4 weeks interval	a, b, d	6 months after last session	Goodman & Baron qualitative global grading; quartile scale for improvement (0–4)
Min (2018) [17]	South Korea	Randomized, split‐face	25	Skin Types III–IV; mild to moderate atrophic acne scars	Fluence: 30–70 mJ/cm^2^; MTZ: 150; spot size: 12 mm; pulse duration: 1 ms; single pass	Double‐spin (details not specified); activated with CaCl_2_; intradermal injection (0.02 mL/site, 1–1.5 cm apart)	Normal saline intradermal	2 sessions; 4‐week interval	b, c, e, f	Up to 84 days after treatment	Investigator's Global Assessment (5‐point); ECCA score; quartile scale
Galal (2019) [18]	Egypt	Randomized, split‐face	30	Skin Types IV–V (70% dark); atrophic acne scars	Power: 15 W; spacing: 800 μm; dwell time: 600 μs; stack: 2	Double‐spin (1200 g/6 min); no activator specified; intradermal injection (“nappage technique”)	Laser alone	3 sessions; 4‐week interval	b	3 months after last session	Goodman & Baron quantitative global scale; Antera 3D image analysis
El‐Hawary (2020) [19]	Egypt	Randomized, comparative (3 groups)	60 (Group 3: *n* = 20)	Skin Types III–IV; atrophic post‐acne scars (moderate to severe)	Power: 12 W; dwell time: 600 μs; spacing: 600 μm; stack: 2	Double‐spin (3000 rpm/7 min → 4000 rpm/5 min); CaCl_2_ activation; intradermal injection (0.1 cc/site, 1 cm apart)	Laser alone (Group 2)	3 sessions; 4‐week interval	a	3 months after last session	Goodman & Baron qualitative global grading; quartile scale (0–4)
Sharma (2021) [20]	India	Randomized, split‐face	30	Grade 3–4 atrophic acne scars (Goodman & Baron); Fitzpatrick Types III–V? (not specified)	Fluence: 25–45 mJ/cm^2^; single pass; stamping mode; PPA: 49	Double‐spin (1000 rpm/10 min → 2000 rpm/5 min); no activator mentioned; intradermal injection (0.2 mL/site, 2 cm apart)	Normal saline intradermal	4 sessions; 4‐week interval	b, c, e, f	2 months after last session	Goodman & Baron qualitative & quantitative scales; patient subjective score (0–10)

*Note:* (a) Excellent improvement rate (> 75% improvement); (b) incidence of hyperpigmentation; (c) incidence of persistent erythema; (d) patient satisfaction rate (very satisfied); (e) erythema duration (days); (f) edema duration (days). For El‐Hawary 2020 (17), only the 20 patients in Group 3 (fractional CO_2_ laser + PRP vs. laser alone) were included in this meta‐analysis; the other 40 patients from Groups 1 and 2 were not included.

### 3.2 Quality Evaluation

**FIGURE 2:**
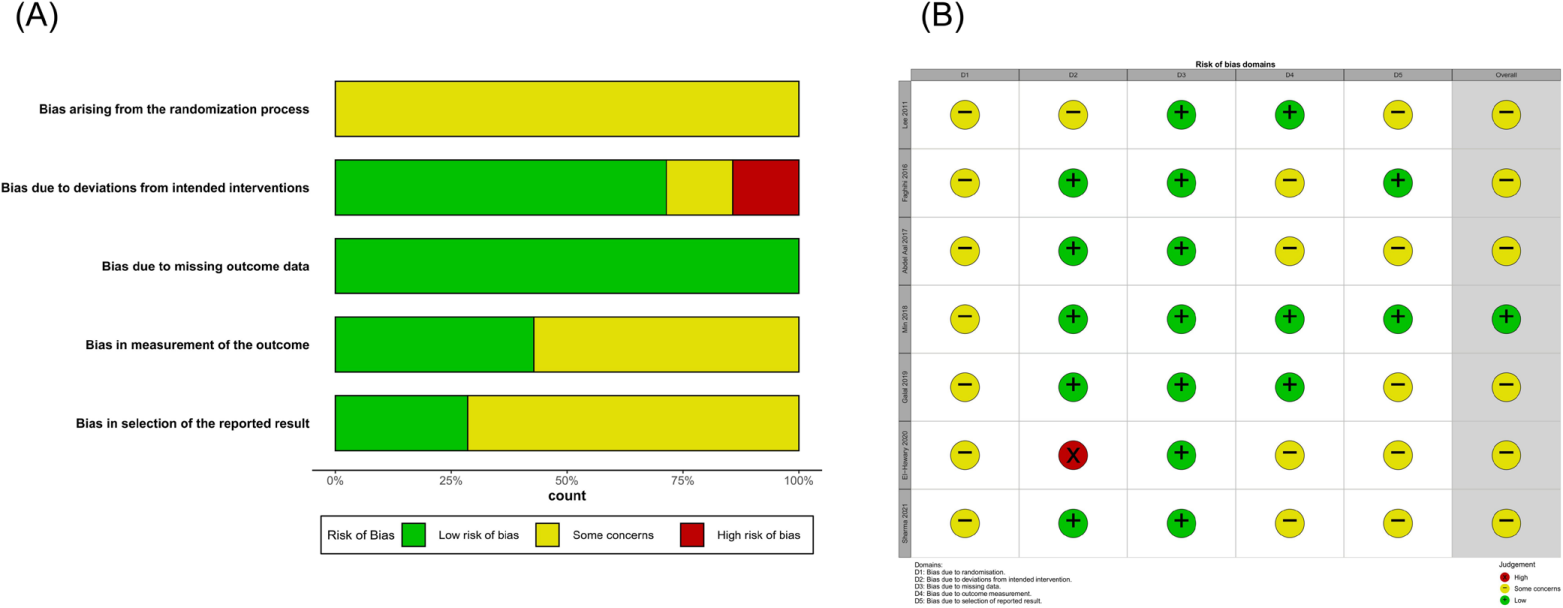
Risk of bias assessment of included randomized controlled trials. (A) Summary plot of risk of bias across studies for each domain. (B) Traffic light plot showing risk of bias assessment for individual studies.

The methodological quality of the seven randomized controlled trials incorporated in this review was assessed using the Cochrane RoB 2.0, and the corresponding findings are illustrated in Figure 2. As illustrated in the summary chart (Figure 2A), all studies raised “some concerns” within the randomization process domain (D1), due to insufficient reporting of sequence generation and allocation concealment. With respect to domain D2, which evaluates protocol adherence and deviations from the assigned interventions, one trial [19] was rated as having a “high risk” of bias due to the lack of blinding among participants and research staff; the remaining investigations received ratings of either “low risk” or “some concerns” in this domain. Notably, all seven studies demonstrated “low risk” of bias for missing outcome data (D3), reflecting adequate participant follow‐up and complete data reporting. In the outcome measurement (D4) domain, half of the studies raised “some concerns”, primarily because patient‐reported outcomes (e.g., satisfaction) were not assessed using blinded methods. Regarding selective reporting of results (D5), most studies raised “some concerns” due to lack of prospective trial registration. Overall, one study [17] was rated “low risk”, while the remaining six studies raised “some concerns” (Figure 2B).

### 3.3 Meta‐Analysis Results

#### 3.3.1 Excellent Improvement Rate

**FIGURE 3:**
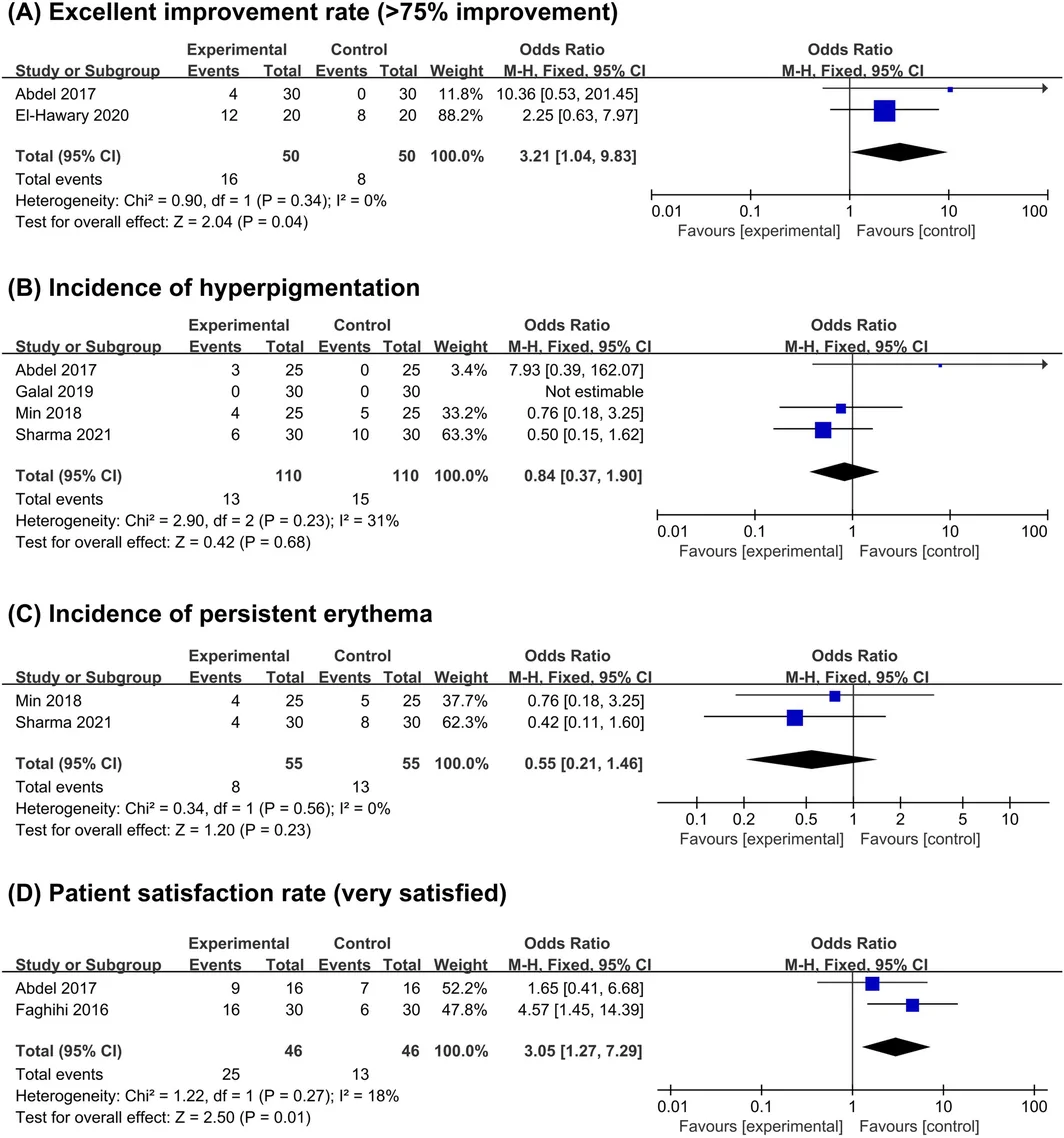
Meta‐analysis of treatment efficacy and selected patient‐reported outcomes. (A) Excellent improvement rate (> 75% improvement). (B) Incidence of hyperpigmentation. (C) Incidence of persistent erythema. (D) Patient satisfaction rate (very satisfied). A fixed‐effect model was applied to all panels because heterogeneity was low (*I* ^2^ < 50% and *p* ≥ 0.10 for all comparisons).

Two studies reported this outcome. Assessment of the data indicated very low heterogeneity between the two studies (*I* ^2^ = 0%, *p* = 0.34), thus allowing for the application of a fixed‐effects model. The aggregated findings revealed that patients receiving combination therapy had a higher rate of excellent improvement than those treated with laser alone (OR = 3.21, 95% CI (1.04–9.83), *p* = 0.04) (Figure 3A).

#### 3.3.2 Incidence of Hyperpigmentation

Four studies reported on the incidence of hyperpigmentation (Figure 3B). The level of heterogeneity detected across the included studies was moderate (*I* ^2^ = 31%, *p* = 0.23); therefore, a fixed‐effects model was applied. Meta‐analysis of the combined data did not demonstrate any statistically significant difference between the two therapeutic approaches (OR = 0.84, 95% CI (0.37–1.90), *p* = 0.68).

#### 3.3.3 Incidence of Persistent Erythema

Data on persistent erythema incidence were available from two studies (Figure 3C). These investigations exhibited no heterogeneity (*I* ^2^ = 0%, *p* = 0.56), allowing for the application of a fixed‐effects model. The pooled estimate revealed no significant difference between the treatment groups (OR = 0.55, 95% CI (0.21, 1.46), *p* = 0.23).

#### 3.3.4 Patient Satisfaction Rate

Two studies reported on the proportion of patients who were “Very Satisfied” (Figure 3D). Low heterogeneity was observed (*I* ^2^ = 18%, *p* = 0.27); therefore a fixed‐effect model was applied. The synthesized results indicated a statistically significant benefit favoring the combination treatment group (OR = 3.05, 95% CI (1.27–7.29), *p* = 0.01).

#### 3.3.5 Erythema Duration

**FIGURE 4:**
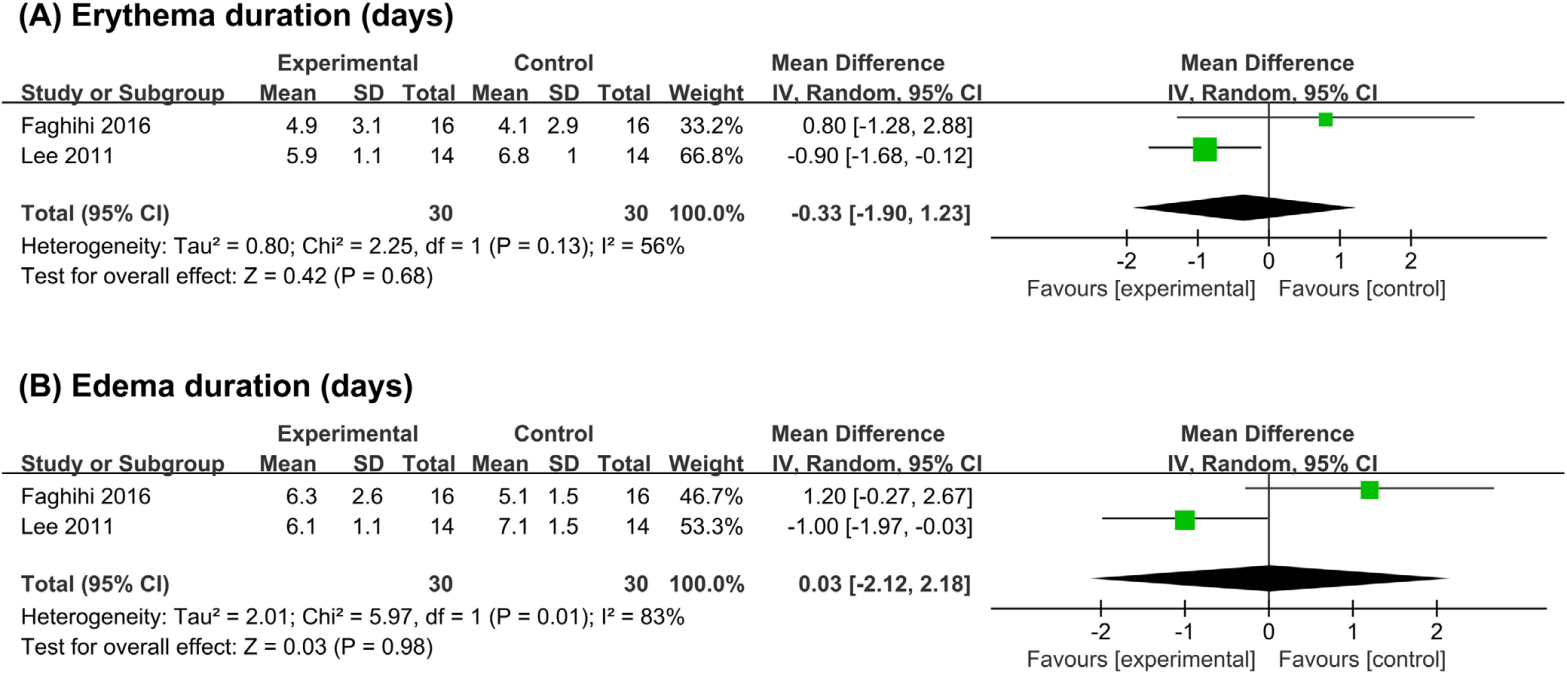
Meta‐analysis of post‐treatment recovery parameters. (A) Forest plot for erythema duration (days). (B) Forest plot for edema duration (days). A random‐effect model was applied to both panels due to substantial heterogeneity (*I* ^2^ > 50%).

Two studies reported on erythema duration (Figure 4A). Examination of the data revealed moderate heterogeneity among the included studies (*I* ^2^ = 56%, *p* = 0.13), leading to the application of a random‐effects model. The pooled meta‐analysis suggested no statistically significant difference when comparing combination therapy with laser monotherapy (MD = −0.33, 95% CI (−1.90, 1.23), *p* = 0.68).

#### 3.3.6 Edema Duration

Two studies reported on edema duration (Figure 4B). A high degree of statistical heterogeneity was observed across the studies (*I* ^2^ = 83%, *p* = 0.01), making the use of a random‐effects model appropriate. The integration of the data revealed no statistically significant difference between the two treatment modalities (MD = 0.03, 95% CI (−2.12, 2.18), *p* = 0.98).

#### 3.3.7 Sensitivity Analysis

Sensitivity analysis for hyperpigmentation by sequentially removing each study [16, 17, 18, 20] produced pooled ORs ranging from 0.59 to 1.43, all with 95% CI that included 1 (e.g., after removing Abdel et al. [16]: OR 0.59 (0.24–1.47); after removing Sharma et al. [20]: OR 1.43 (0.44–4.69)). Heterogeneity varied (*I* ^2^ = 0%–66%), but no single study altered the overall conclusion of no significant difference between treatments.

## 4 Discussion

### 4.1 Principal Findings

By aggregating evidence from available randomized controlled trials, this study confirms that combining autologous platelet‐rich plasma with fractional CO_2_ laser therapy is more effective than laser treatment alone for atrophic acne scars. In particular, the combined approach was linked to statistically significant improvements in both the percentage of individuals achieving an excellent clinical outcome (exceeding 75% improvement; OR = 3.21, 95% CI: 1.04–9.83) and the number reporting themselves as “very satisfied” with the results (OR = 3.05, 95% CI: 1.27–7.29). Therefore, for patients seeking a higher probability of clinical improvement and greater satisfaction, combination therapy may serve as a preferred clinical option.

### 4.2 Biological Rationale

The findings align with the theoretical mechanism of combined therapy. Fractional CO_2_ laser creates ordered microthermal injury zones through focal photothermal effects, activating collagen remodeling repair pathways while simultaneously enhancing transdermal delivery efficiency via the generated microchannels [6]. The microchannels generated through fractional laser ablation act as pathways that facilitate the transport of PRP's diverse growth factors—such as platelet‐derived growth factor (PDGF), transforming growth factor‐beta (TGF‐β), vascular endothelial growth factor (VEGF), and insulin‐like growth factor‐1 (IGF‐1)—into the dermal layer. Once localized in the dermis, these bioactive molecules exert pivotal regulatory influence over the early stages of tissue repair. These effects include mitigating excessive inflammation, promoting angiogenesis, and continuously stimulating fibroblast collagen synthesis, ultimately achieving higher‐quality tissue regeneration and collagen deposition [7, 17].

### 4.3 Heterogeneity and Influencing Factors

However, this study also noted heterogeneity among different outcome measures. Although combination therapy showed a significant advantage in the proportion of patients reporting “very satisfied”, the heterogeneity was low (*I* ^2^ = 18%). The effect size for excellent improvement appeared to be influenced by a single study; when El‐Hawary et al. [19] (the only study with a high risk of bias in one domain) was excluded, the pooled OR rose from 3.21 to 10.75. This shift may be linked to the study's methodological profile—despite its higher risk of bias from non‐blinding, the incorporation of histopathological measures and three treatment rounds likely contributed to a more conservative assessment of clinical outcomes. Heterogeneity in patient satisfaction likely stems from differences across studies in assessment tools, timing, and patient perception of adverse effects. For example, Abdel Aal et al. [16] documented greater satisfaction levels at the six‐month follow‐up visit. In contrast, Faghihi et al. [15] observed no statistically significant difference between groups, a finding potentially associated with more intense erythema and edema subjectively reported by individuals in the combined treatment arm. These variations suggest that while combination therapy significantly increases the likelihood of achieving top‐tier satisfaction, the precise rate remains influenced by study design and assessment methodologies—factors that should be considered during clinical discussions between physicians and patients.

### 4.4 Safety Profile

From a safety standpoint, this study demonstrated that adding PRP did not significantly reduce the duration of erythema or edema, nor did it significantly lower the incidence of post‐inflammatory hyperpigmentation [21]. This suggests that PRP's “repair‐optimizing” effect may primarily manifest in enhancing healing quality and long‐term structural remodeling, while its regulatory impact on the acute phase response—characterized by vasodilation and inflammatory cell infiltration directly induced by laser treatment—may be limited [6, 22].

### 4.5 Impact of Technical Variations

Notably, despite all studies employing combined strategies, variations in intervention details remain a significant source of heterogeneity. For instance, PRP preparation methods (centrifugation parameters, activator usage), final form (liquid or gel), and laser parameters (energy density, coverage) were not standardized across studies [8, 23]. Existing evidence indicates that PRP obtained through different preparation methods exhibits substantial variations in platelet concentration, growth factor release kinetics, and the presence of leukocytes [24, 25]. Specifically, double‐spin centrifugation yields higher platelet enrichment than single‐spin, but may also concentrate pro‐inflammatory cells if not carefully controlled [25]. Activation methods (e.g., calcium chloride, thrombin, or mechanical activation) further influence the timing and profile of growth factor release, potentially affecting clinical outcomes [24]. The gel form, while offering better local retention, might alter the diffusion of bioactive molecules compared with the liquid form [8]. Similarly, variations in laser parameters (energy density, coverage density, number of passes) directly regulate the depth and intensity of dermal injury, thereby modulating the synergistic interaction with PRP. This lack of protocol standardization represents a major limitation in the current evidence base, as it precludes the development of definitive clinical recommendations. A significant gap stems from the inability to conduct thorough subgroup analyses on these important technical factors, a constraint imposed by the limited number of studies available for inclusion [24, 25].

### 4.6 Limitations

When interpreting the outcomes of this study, several limitations must be taken into account. First, the limited number of randomized controlled trials included and the relatively modest total sample size restricted the statistical power and hindered a thorough evaluation of potential publication bias. Second, the methodological quality of the original studies was frequently inadequate, as the majority failed to provide sufficient details regarding random sequence generation, allocation concealment, and blinding procedures. This may introduce implementation and measurement bias, particularly in the assessment of patient‐reported outcomes [26]. Third, significant clinical heterogeneity exists, with substantial variations among studies in key intervention details such as PRP preparation techniques, administration routes, and laser parameters [8, 27]. This not only constitutes a primary cause of outcome heterogeneity but also hinders the development of standardized recommendations for clinical practice. Fourth, follow‐up periods in all studies did not exceed 6 months, precluding assessment of long‐term efficacy maintenance, recurrence risk, and delayed adverse reactions. Finally, study populations predominantly featured Middle Eastern and East Asian skin tones (Fitzpatrick Types III–V), with insufficient data on fair‐skinned (Types I–II) and dark‐skinned (Type VI) individuals, limiting the generalizability of conclusions. Sixth, although six of the seven included trials employed a split‐face design, none of those six reported individual patient‐level paired data. The remaining trial [19] used a three‐arm parallel design. Therefore, we were unable to perform a paired meta‐analysis that accounts for within‐patient correlation. Treating split‐face data as independent groups may underestimate variance and narrow confidence intervals, although the consistent direction of effect across sensitivity analyses supports the robustness of our findings. Seventh, only studies published in English were included, which may introduce language bias and limit the generalizability of the findings to non‐English literature.

## 5 Conclusion

In conclusion, the available evidence suggests that fractional CO_2_ laser combined with autologous PRP may offer short‐term efficacy and patient satisfaction benefits over laser monotherapy for atrophic acne scars, with a comparable safety profile. However, these findings are preliminary due to the small sample size, low heterogeneity in patient satisfaction, and methodological limitations of the primary trials, including short follow‐up (≤ 6 months in all included studies) and a study population predominantly restricted to Middle Eastern and East Asian skin types (Fitzpatrick III–V), which limit the generalizability of our conclusions. Future large‐scale, multicenter, methodologically rigorous randomized controlled trials with extended follow‐up (≥ 12 months), inclusion of broader skin types (I–II and VI), and standardized PRP preparation protocols are needed to confirm these observations and guide clinical practice.

## Author Contributions

Qiang Hu: conceptualization, methodology, formal analysis, writing – original draft, project administration. Shuang Sui: investigation, data curation, validation, writing – review and editing. Zhenghua Yang: literature search, screening, data extraction, risk of bias assessment. Kaiwen Zhang: statistical analysis, visualization, writing – review and editing. Haibo Wang: supervision, resources, methodology, writing – review and editing.

## Ethics Statement

As a systematic review, this study synthesizes data from previously published trials and does not involve primary data collection from human participants. Ethical approval and informed consent were obtained in each original included study.

## Consent

The authors have nothing to report.

## Conflicts of Interest

The authors declare no conflicts of interest.

## Supporting information


**Appendix S1:** Detailed search strategies for each database.

## Data Availability

All data analyzed in this study are included in the cited references.

## References

[1] T. Jennings , R. Duffy , M. McLarney , et al., “Acne Scarring—Pathophysiology, Diagnosis, Prevention and Education: Part I,” Journal of the American Academy of Dermatology 90, no. 6 (2024): 1123–1134, https://www.ncbi.nlm.nih.gov/pubmed/35792196. 35792196 10.1016/j.jaad.2022.04.021

[2] X. Pang , H. Chi , Z. Zhan , Z. Yu , and M. Cai , “CO_2_ Laser Treatment for Scars After Cleft Lip Surgery: A Systematic Review and Meta‐Analysis,” BMC Oral Health 24, no. 1 (2024): 1443, https://www.ncbi.nlm.nih.gov/pubmed/39604962. 39604962 10.1186/s12903-024-05205-6 PMC11603913

[3] R. Leszczynski , C. A. da Silva , C. P. N. Pinto , U. Kuczynski , and E. M. K. da Silva , “Laser Therapy for Treating Hypertrophic and Keloid Scars,” Cochrane Database of Systematic Reviews 9 (2022): CD011642, https://www.ncbi.nlm.nih.gov/pubmed/36161591. 36161591 10.1002/14651858.CD011642.pub2 PMC9511989

[4] C. Xiao , J. Zhang , and J. Peng , “Long‐Term Efficacy and Safety of Carbon Dioxide (CO(2)) Array Laser Versus Erbium‐Doped Yttrium Aluminum Garnet (Er:YAG) Laser for Acne Scars,” American Journal of Translational Research 17, no. 8 (2025): 6391–6402, https://www.ncbi.nlm.nih.gov/pubmed/40950268. 40950268 10.62347/WTMW1778 PMC12432690

[5] C. Long , Y. Xu , Q. Lin , et al., “Efficacy of Fractional Laser Combined With Bipolar Radiofrequency in the Treatment of Atrophic Facial Acne Scaring,” American Journal of Translational Research 17, no. 1 (2025): 116–124, https://www.ncbi.nlm.nih.gov/pubmed/39959212. 39959212 10.62347/PQOJ2511 PMC11826168

[6] S. I. Gaumond , R. Abdin , M. Yaghi , et al., “Platelet‐Rich Plasma as an Adjuvant Therapy to Fractional Ablative Carbon Dioxide Lasers for Cutaneous Repair: A Complementary Treatment for Atrophic Acne Scarring,” Lasers in Medical Science 39, no. 1 (2024): 254, https://www.ncbi.nlm.nih.gov/pubmed/39387947. 39387947 10.1007/s10103-024-04192-y PMC11467096

[7] J. Chen , Y. Wan , Y. Lin , and H. Jiang , “Fractional Carbon Dioxide Laser or Erbium:Yttrium‐Aluminum‐Garnet Laser Assisted by Topical Application/Intradermal Injection of Platelet‐Rich Plasma for Postacne Scars,” Plastic and Reconstructive Surgery 148, no. 6 (2021): 915e–927e, https://www.ncbi.nlm.nih.gov/pubmed/34847111. 10.1097/PRS.0000000000008513 34847111

[8] H. I. Gawdat , Y. A. El‐Hadidy , R. S. H. M. Allam , and H. A. Abdelkader , “Autologous Platelet‐Rich Plasma ‘Fluid’ Versus ‘Gel’ Form in Combination With Fractional CO_2_ Laser in the Treatment of Atrophic Acne Scars: A Split‐Face Randomized Clinical Trial,” Journal of Dermatological Treatment 33, no. 5 (2022): 2654–2663, https://www.ncbi.nlm.nih.gov/pubmed/35435087. 35435087 10.1080/09546634.2022.2067816

[9] Y. E. Aljefri , A. A. Ghaddaf , R. A. Alahmadi , et al., “Ablative Fractional Carbon Dioxide Laser Combined With Autologous Platelet‐Rich Plasma in the Treatment of Atrophic Acne Scars: A Systematic Review and Meta‐Analysis,” Dermatologic Therapy 35, no. 12 (2022): e15888, https://www.ncbi.nlm.nih.gov/pubmed/36183145. 36183145 10.1111/dth.15888

[10] N. Wu , H. Sun , Q. Sun , et al., “A Meta‐Analysis of Fractional CO_2_ Laser Combined With PRP in the Treatment of Acne Scar,” Lasers in Medical Science 36, no. 1 (2020): 1–12, https://www.ncbi.nlm.nih.gov/pubmed/32827074. 32827074 10.1007/s10103-020-03105-z

[11] M. Roohaninasab , A. Jafarzadeh , S. Zare , et al., “Evaluation of the Efficacy, Safety, and Satisfaction Rates of Platelet‐Rich Plasma, Non‐Cross‐Linked Hyaluronic Acid, and Their Combination in Patients With Acne Scars Treated With Fractional CO_2_ Laser: A Randomized, Double‐Blind, Split‐Face Comparative Study,” Aesthetic Plastic Surgery 50, no. 3 (2025): 1277–1290, https://www.ncbi.nlm.nih.gov/pubmed/41168367. 41168367 10.1007/s00266-025-05359-w

[12] S. Arora , S. Godara , R. Dabas , G. Arora , G. Renganathan , and R. Choudhary , “A Comparative Study on the Efficacy of Fractional CO_2_ Laser and Fractional CO_2_ Laser With Autologous Platelet‐Rich Plasma in Scars,” Indian Dermatology Online Journal 11, no. 6 (2020): 930, https://www.ncbi.nlm.nih.gov/pubmed/33344342. 33344342 10.4103/idoj.IDOJ_174_20 PMC7734978

[13] D. Moher , A. Liberati , J. Tetzlaff , D. G. Altman , and PRISMA Group , “Preferred Reporting Items for Systematic Reviews and Meta‐Analyses: The PRISMA Statement,” Annals of Internal Medicine 151, no. 4 (2009): 264–269, https://www.ncbi.nlm.nih.gov/pubmed/19622511. 19622511 10.7326/0003-4819-151-4-200908180-00135

[14] J. W. Lee , B. J. Kim , M. N. Kim , and S. K. Mun , “The Efficacy of Autologous Platelet Rich Plasma Combined With Ablative Carbon Dioxide Fractional Resurfacing for Acne Scars: A Simultaneous Split‐Face Trial,” Dermatologic Surgery 37, no. 7 (2011): 931–938, https://www.ncbi.nlm.nih.gov/pubmed/21635618. 21635618 10.1111/j.1524-4725.2011.01999.x

[15] G. Faghihi , S. Keyvan , A. Asilian , S. Nouraei , S. Behfar , and M. Nilforoushzadeh , “Efficacy of Autologous Platelet‐Rich Plasma Combined With Fractional Ablative Carbon Dioxide Resurfacing Laser in Treatment of Facial Atrophic Acne Scars: A Split‐Face Randomized Clinical Trial,” Indian Journal of Dermatology, Venereology and Leprology 82, no. 2 (2016): 162, https://www.ncbi.nlm.nih.gov/pubmed/26924405. 26924405 10.4103/0378-6323.174378

[16] A. M. Abdel Aal , I. M. Ibrahim , N. A. Sami , and I. M. Abdel Kareem , “Evaluation of Autologous Platelet‐Rich Plasma Plus Ablative Carbon Dioxide Fractional Laser in the Treatment of Acne Scars,” Journal of Cosmetic and Laser Therapy 20, no. 2 (2017): 106–113, https://www.ncbi.nlm.nih.gov/pubmed/28853968. 28853968 10.1080/14764172.2017.1368667

[17] S. min , J. Y. Yoon , S. Y. Park , J. Moon , H. H. Kwon , and D. H. Suh , “Combination of Platelet Rich Plasma in Fractional Carbon Dioxide Laser Treatment Increased Clinical Efficacy of for Acne Scar by Enhancement of Collagen Production and Modulation of Laser‐Induced Inflammation,” Lasers in Surgery and Medicine 50, no. 4 (2018): 302–310, https://www.ncbi.nlm.nih.gov/pubmed/29266290. 29266290 10.1002/lsm.22776

[18] O. Galal , A. A. Tawfik , N. Abdalla , and M. Soliman , “Fractional CO_2_ Laser Versus Combined Platelet‐Rich Plasma and Fractional CO_2_ Laser in Treatment of Acne Scars: Image Analysis System Evaluation,” Journal of Cosmetic Dermatology 18, no. 6 (2019): 1665–1671, https://www.ncbi.nlm.nih.gov/pubmed/30964227. 30964227 10.1111/jocd.12909

[19] E. E. El‐Hawary , S. Nassar , A. A. Hodeib , M. M. Shareef , and M. M. Fawzy , “Ablative Fractional Carbon Dioxide Laser and Autologous Platelet‐Rich Plasma in the Treatment of Atrophic Acne Scars: A Comparative Clinico‐Immuno‐Histopathological Study,” Lasers in Surgery and Medicine 53, no. 4 (2020): 458–467, https://www.ncbi.nlm.nih.gov/pubmed/32770685. 32770685 10.1002/lsm.23306

[20] S. Sharma , J. Kaur , T. Kaur , and R. Bassi , “Fractional Carbon Dioxide Laser Versus Combined Fractional Carbon Dioxide Laser With Platelet‐Rich Plasma in the Treatment of Atrophic Post‐Acne Scars: A Split‐Face Comparative Study,” Journal of Cutaneous and Aesthetic Surgery 14, no. 1 (2021): 41, https://www.ncbi.nlm.nih.gov/pubmed/34084007. 34084007 10.4103/JCAS.JCAS_147_19 PMC8149982

[21] B. Yao , T. Chen , Y. Zhang , L. Zhang , M. Cai , and X. Yu , “Clinical Characteristics of Patients Receiving Fractional Laser Therapy for Facial Scars and Their Association With the Duration of Postoperative Erythema,” American Journal of Translational Research 17, no. 11 (2025): 8665–8674, https://www.ncbi.nlm.nih.gov/pubmed/41415063. 41415063 10.62347/UGRI6193 PMC12709358

[22] J.‐I. Na , J.‐W. Choi , H.‐R. Choi , et al., “Rapid Healing and Reduced Erythema After Ablative Fractional Carbon Dioxide Laser Resurfacing Combined With the Application of Autologous Platelet‐Rich Plasma,” Dermatologic Surgery 37, no. 4 (2011): 463–468, https://www.ncbi.nlm.nih.gov/pubmed/21481064. 21481064 10.1111/j.1524-4725.2011.01916.x

[23] M. A. Rageh , A. A. Tawfik , N. Abdallah , and S. M. A. Ibrahim , “Fractional CO_2_ Laser Combined With Autologous Nanofat Injection Versus Fractional CO_2_ Laser Combined With Platelet‐Rich Plasma in the Treatment of Atrophic Acne Scars: A Split‐Face Comparative Study With Optical Skin Imaging,” Dermatologic Surgery 50, no. 1 (2024): 75–80, https://www.ncbi.nlm.nih.gov/pubmed/38048184. 38048184 10.1097/DSS.0000000000003968

[24] E. Ragni , M. M. Taiana , T. Cengic , L. de Girolamo , and M. Ostojic , “PRP or Not PRP: Is the Debate Surrounding Platelets‐Based Blood‐Derived Products Evolving?,” Knee Surgery, Sports Traumatology, Arthroscopy 33, no. 5 (2025): 1920–1924, https://www.ncbi.nlm.nih.gov/pubmed/40079361. 10.1002/ksa.12655 PMC12022825 40079361

[25] C. F. Mourao and G. G. Alves , “The Role of Platelet Derivatives in Regenerative Medicine: Biological Foundations and Translational Perspectives,” Frontiers in Cell and Developmental Biology 14 (2026): 1770953, https://www.ncbi.nlm.nih.gov/pubmed/41988383. 41988383 10.3389/fcell.2026.1770953 PMC13076266

[26] J. A. C. Sterne , J. Savović , M. J. Page , et al., “RoB 2: A Revised Tool for Assessing Risk of Bias in Randomised Trials,” BMJ 366 (2019): l4898, https://www.ncbi.nlm.nih.gov/pubmed/31462531. 31462531 10.1136/bmj.l4898

[27] C. G. Manole , C. Soare , L. C. Ceafalan , and V. M. Voiculescu , “Platelet‐Rich Plasma in Dermatology: New Insights on the Cellular Mechanism of Skin Repair and Regeneration,” Life 14, no. 1 (2023): 40, https://www.ncbi.nlm.nih.gov/pubmed/38255655. 38255655 10.3390/life14010040 PMC10817627

