# Supplementary material for: Adjunctive Use of Platelet‐Rich Plasma With Fractional CO2 Laser Versus Standalone Laser Therapy for the Treatment of Atrophic Acne Scarring: A Systematic Review and Meta‐Analysis
Source: J Cosmet Dermatol. 2026 Sep 10;25(9):e71141. doi: 10.1111/jocd.71141 (PMC13562814; doi:10.1111/jocd.71141)
Supplement: Supplementary file 1 [file JOCD-25-e71141-s001.docx]

**Appendix S1: Detailed Search Strategies for Each Database**

**Search date: July 31, 2025**

**1. PubMed**

("Platelet-Rich Plasma"[Mesh] OR "platelet rich plasma"[tiab] OR PRP[tiab])

AND ("Acne Vulgaris"[Mesh] OR acne[tiab])

AND ("Cicatrix"[Mesh] OR scar[tiab] OR "atrophic scar"[tiab])

AND ("Lasers, Gas"[Mesh] OR "Carbon Dioxide"[Mesh] OR "fractional CO2 laser"[tiab] OR "CO2 laser"[tiab])

AND ("Randomized Controlled Trial"[pt] OR randomized[tiab] OR randomised[tiab] OR placebo[tiab])

**2. Embase**

('platelet rich plasma' OR 'platelet-rich plasma' OR 'thrombocyte rich plasma')

AND ('cicatrices' OR 'cicatrix' OR 'radiation scar' OR 'scar')

AND ('Acupulse' OR 'carbon dioxide laser device' OR 'carbon dioxide laser device (physical object)' OR 'carbon dioxide laser systems' OR 'co 2 laser' OR 'CO2 laser' OR 'continuous wave carbon dioxide laser' OR 'Ds-40u' OR 'eCO2' OR 'Fraxel Re:fine' OR 'general use solid state carbon dioxide laser' OR 'general/multiple surgical solid-state/carbon dioxide laser system' OR 'Juvia' OR 'laser, carbon dioxide' OR 'laser, continuous wave carbon dioxide' OR 'Lasertronics Paragon 50' OR 'Mosaic CO2' OR 'Opelaser-03 S' OR 'Pixel Perfect' OR 'SCx10 Carbon Dioxide Slab Laser' OR 'Smartxide' OR 'SmartXide DOT' OR 'solid state carbon dioxide laser system' OR 'UltraPulse Encore' OR 'UltraPulse Surgitouch' OR 'UM-L30' OR 'carbon dioxide laser')

AND ('controlled trial, randomized' OR 'randomised controlled study' OR 'randomised controlled trial' OR 'randomized controlled study' OR 'trial, randomized controlled' OR 'randomized controlled trial')

**3. Web of Science**

TS=("Platelet-Rich Plasma" OR "platelet rich plasma" OR PRP)

AND TS=(acne)

AND TS=("cicatrix" OR scar OR "atrophic scar")

AND TS=("fractional CO2 laser" OR "CO2 laser" OR "carbon dioxide laser")

AND TS=(randomized OR randomised OR "randomized controlled trial" OR RCT OR placebo)

**4. Cochrane Library**

#1 MeSH descriptor: [Platelet-Rich Plasma] explode all trees

#2 (platelet rich plasma):ti,ab,kw OR PRP:ti,ab,kw

#3 #1 OR #2

#4 MeSH descriptor: [Cicatrix] explode all trees

#5 (scar OR scarring OR atrophic scar):ti,ab,kw

#6 #4 OR #5

#7 MeSH descriptor: [Lasers, Gas] explode all trees

#8 (fractional CO2 laser OR CO2 laser OR carbon dioxide laser):ti,ab,kw

#9 #7 OR #8

#10 (randomized OR randomised OR placebo):ti,ab,kw

#11 #3 AND #6 AND #9 AND #10
